# Magnetic resonance imaging and mammographic appearance of dermatofibrosarcoma protuberans in a male breast: a case report and literature review

**DOI:** 10.4076/1752-1947-3-8246

**Published:** 2009-06-16

**Authors:** Xin Chen, Yung Hsin Chen, Yi-li Zhang, You-min Guo, Zhi-lan Bai, Xian Zhao

**Affiliations:** 1 Department of Radiology, Second Hospital of Medical College of Xi'an Jiaotong University Xi'an, Shaanxi, China; 2 Department of Radiology, Good Samaritan Hospital, Brockton, MA, USA; 3 Department of Radiology, First Hospital of Medical College of Xi'an Jiaotong University Xi'an, Shaanxi, China

## Abstract

**Introduction:**

Dermatofibrosarcoma protuberans is a rare low-grade soft tissue neoplasm with trunk and extremities being the most common sites of involvement. We report a rare case of male breast with dermatofibrosarcoma protuberans and its imaging features. To our knowledge the imaging appearance of dermatofibrosarcoma protuberans of the breast has never been reported in the literature.

**Case presentation:**

We report the imaging appearance of dermatofibrosarcoma protuberans on the breast of a 41-year-old Chinese man who initially presented with a palpable lump. A mammogram showed two lesions, one with well circumscribed and the other with an ill defined border, in his right breast. Conventional magnetic resonance imaging was performed and showed the well defined larger lesion with mild central hypointensity while the smaller lesion had an irregular border. Both lesions were well characterized on the fat-suppressed sequences.

**Conclusions:**

Dermatofibrosarcoma protuberans is a rare soft tissue sarcoma and its occurrence on the breast is even rarer. Mammography and magnetic resonance imaging can help in characterizing the lesion and localizing the lesion for further diagnostic evaluation and surgical planning.

## Introduction

Dermatofibrosarcoma protuberans (DFSP) is a rare low-grade soft tissue neoplasm that originates from the dermis and accounts for about 1.8% of all soft tissue sarcomas [1,2]. The tumor may occur on any part of the body with the trunk and extremities being the most common sites of involvement where the incidence is 47% and 38% respectively [3]. However, the occurrence of dermatofibrosarcoma protuberans on the breast is rare. Here we present a case of dermatofibrosarcoma protuberans on the breast of a Chinese man. Diagnostic work-up comprised clinical examination, mammography and magnetic resonance imaging (MRI), and diagnosis was proved by both pathology and immunohistochemistry through surgical excision. To our knowledge, this is the first report of the imaging appearance of dermatofibrosarcoma protuberans of the breast.

## Case presentation

A 41-year-old Chinese man presented with palpable right breast lumps for 1 year. On physical examination, a large retroareolar hard nontender mass approximately 4.5 cm in size was palpated over the right breast. The mass was nonmobile and superficial but did not have any overlying skin findings. A smaller adjacent mobile and nontender lesion, approximately 3 cm, was palpated just medial to the first mass. Initial laboratory evaluation was normal for prolactin, estradiol, luteinizing and follicle-stimulating hormone levels. Furthermore, he had never previously been treated with radiation, according to his past medical history.

**Figure 1:**
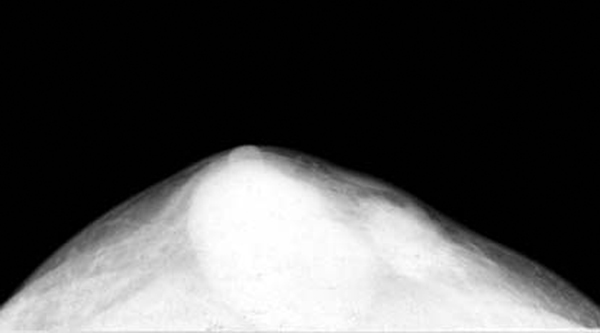
**Axial mammography shows a subcutaneous oval mass with a smooth and sharp margin on the right breast, and another smaller lesion with a less well-defined margin is seen adjacent to the main lesion**.

Conventional mammography was performed on Flat III (Metaltronica Company, Rome, Italy). Mammography showed a subcutaneous oval mass with a smooth and sharp margin on his right breast, and another small oval mass with a less well-defined margin was seen adjacent to the main lesion (Figure 1).

**Figure 2:**
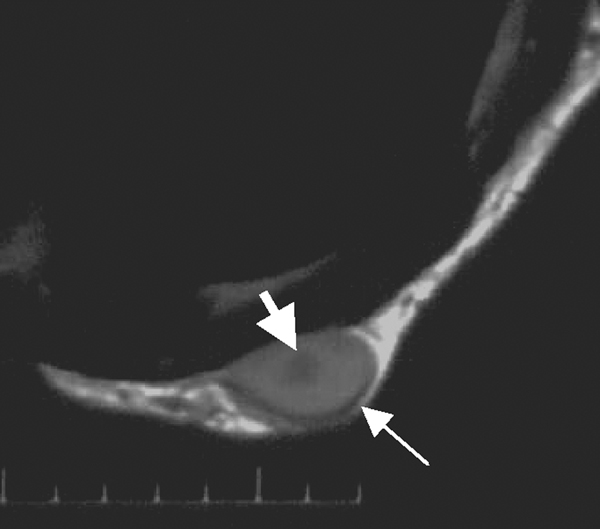
**Axial T2-weighted magnetic resonance image shows a heterogeneous low signal mass with a low signal central region (upper arrow) and between the edge of which and the surrounding fat tissue there is a district rim with low signal intensity (lower arrow)**.

**Figure 3:**
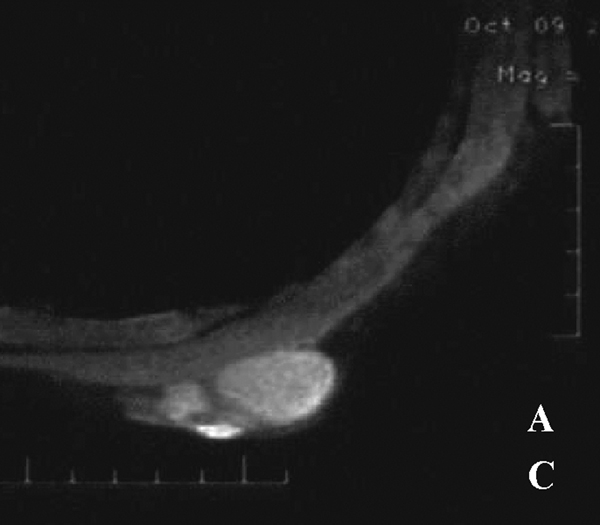
**Axial fat-suppressed T1-weighted image shows two lesions clearly, and the signal intensity of both lesions is higher than that of the pectoralis major muscle**.

MR imaging was performed on a 1.5 T Signa Infinity TwinSpeed MR scanner (GE Company, Milwaukee, WI, USA). The examination comprised of routine T1- and T2-weighted fast spin echo (FSE) sequences in axial and sagittal planes; T1- and T2-weighted imaging fat-suppressed in axial and sagittal planes, respectively. On T1-weighted imaging, the lesions were predominantly hypointense to subcutaneous fat and mildly hyperintense to the pectoralis major muscle. On T2-weighted imaging, the lesions were of a higher signal than the subcutaneous fat. Furthermore, the larger lesion had a smooth contour and well defined borders on all sequences which, on T2-weighted images, had a lower signal central region. Along the border of the larger lesion, there was a distinct rim of decrease in signal between the lesion and fat interface (Figure 2), whereas, the small lesion had a less poorly defined border on conventional T2 weighted images. On the fat-suppressed sequences, both lesions had better depiction for the margins and borders and there was a mild mass effect of the dominant lesion on the underlying pectoralis major muscle (Figure 3).

The gross pathology of the specimens after surgery showed a yellowish tanned smooth surface mass measuring 4.5 cm × 3.0 cm × 2.0 cm and a smaller lesion with similar features measuring 2.5 cm × 2.0 m × 1.0 cm. Histologic specimens showed high cellularity monomorphic slender spindle cells arrayed in a storiform pattern aligned at right angles to small vessels and collagen fibers intermixed with scattered adipose tissue. The nuclei of the spindle cells were well differentiated with only rare mitotic figures. The larger mass had a fibrous envelope and plentiful collagen fibers in the central region. The smaller mass lacked the fibrous envelope. There was positive immunohistologic staining for vimentin and cluster of differentiation (CD) 34, negative for b-cell leukemia-lymphoma (Bcl -2), S-100 protein, smooth muscle actin (SMA), desmin and epithelial membrane antigen (EMA).

## Discussion

DFSP was first described by Taylor in 1890, and was described as a gradually and recurrent cutaneous neoplasm in 1924 by Darier and Ferrand. The term "dermatofibrosarcoma protuberans" was coined by Hoffman in 1925. It is a low degree malignant soft tissue tumor typically arising in the dermis, which then spreads into the subcutaneous tissues and muscle [4]. Males are slighted more commonly affected than females and the male-to-female ratio is approximately 3:2. The tumor occurs in patients of all ages, with the highest frequency occurring in the fourth decade of life [5,6]. The trunk and extremities are the most common sites of involvement, accounting for almost 85% of all cases [3]. However, its occurrence on the breast is very rare.

Histologically DFSP is distinctively composed by monomorphic spindle cells arrayed in a matter or storiform pattern and a positive CD34 is a help in diagnosis [6].

Clinically DFSP tends to exhibit an indolent growth pattern and is usually less than 5 cm in size [7]. In addition, DFSPs are superficial in 77% of patients and, according to the report of Bowne et al, had invaded deeper structures in only 22% of patients [7]. The lesions arise as pink or violet-red plaques, and the surrounding skin may be telangiectatic [8]. Tumors, generally, are fixed to the dermis but move freely over deeper-lying tissue, but often fixed to more deeply seated structures in advanced and/or recurrent cases [8].

DFSP is characterized by local invasion and recurrence. Metastases, however, are rare and large excisions are necessary to reduce the risk of recurrence. The likelihood of local recurrence after this procedure is performed is less than 10% [8]; in contrast, the risk of local recurrence exceeds 50% when the final margins are positive [9].

Most DFSPs are typically small and superficial and diagnosis may be suspected on the basis of the tumor's clinical appearance and pathologic examination. Consequently, they usually are not imaged. When the tumor is large, particularly a large recurrence lesion, magnetic resonance imaging (MRI) is useful for ascertaining the depth of tumor invasion. In addition, MRI is a help in evaluating position and a differential diagnosis for a tumor which occurs in an atypical site. Up to now, Kransdorf and Meis-Kindbom reported the MR imaging appearances of eleven cases [4]; Torreggiani reported the MR imaging findings of ten patients [10], however, among which there is no reported case of breast DFSPs in the literature. According to their reports, the MR appearance of DFSP was a well-defined lesion that had prolonged T1 and T2 relaxation times [4]. On T1-weighted imaging, the tumor was isointense, slightly hypointense or hyperintense to skeletal muscle. Compared with that of fat, the tumor was of a high or intermediate signal, however a border can be hard to separate from fat on conventional T2-weighted images without fat saturation, but better depicted on short tau inversion recovery (STIR) imaging with the signal approaching water or blood. Enhancement can be variable due to levels of necrosis or hemorrhage [10].

Our case occurred on a male breast. The larger lesion appeared as a hard mass which adhered to the skin and was immobile, while the smaller lesion was located within subcutaneous tissue and was mobile and more characteristic on physical examination. Because benign hypertrophy and breast cancer are the two most common male breast diseases, the primary clinical diagnosis was therefore breast cancer and mammography and MR imaging were arranged for the patient. Both the larger and the smaller masses were displayed clearly on mammography films. In contradistinction to breast cancer, the larger mass transfixed the subcutaneous fat and depressed the underlying pectoralis muscle. Furthermore, the larger mass manifested the features of a benign mass with a regular shape and a well-defined and clear margin. The lesions' signal intensity on conventional FSE sequence was similar to the literature, which was hypointense to subcutaneous fat and mildly hyperintense to the pectoralis major muscle on T1-weighted imaging; and hyperintense to subcutaneous fat on T2-weighted imaging. Interestingly the larger lesion had a distinct low signal rim along its margin and a lower signal center of T2-weighted imaging. Histologic specimens showed it had a fibrous envelope and plentiful collagen fibers in the central region. Compared with Torreggiani's report, we used the saturation method of frequency selection to perform fat-suppressed imaging which is convenient and easy to use, and possesses good specificity to suppress the fat signal [10]. Both lesions were a slightly higher signal to the muscle on T1-weighted fat-suppressed sequences and significantly hyperintense on T2-weighted. In particular, the smaller lesion showed more defined margins than routine sequences by high-signal fat tissue being suppressed, which is useful for accurate preoperative assessment.

## Conclusions

DFSP is a rare soft tissue sarcoma, most of which do not require radiologic evaluation. However, if the tumor occurs on the breast, mammography and MR imaging may be necessary for its localization and differential diagnosis. When the tumor locates inside the subcutaneous fat with prolonged T1 and T2 relaxation times and a well-defined margin, and exhibits an indolent growth pattern and skin plaques, DFSP should be considered. Large excision should be performed to reduce the risk of recurrence.

## Abbreviations

CD: cluster of differentiation; DFSP: Dermatofibrosarcoma protuberans; EMA: epithelial membrane antigen; FSE: fast spin echo; MRI: magnetic resonance imaging; SMA: smooth muscle actin; STIR: short tau inversion recovery.

## Consent

Written informed consent was obtained from the patient for publication of this case report and accompanying images. A copy of the written consent is available for review by the Editor-in-Chief of this journal.

## Competing interests

The authors declare that they have no competing interests.

## Authors' contributions

CX and GYM were involved in patient management and writing of the manuscript. CYH and ZYL were involved in the writing of the manuscript. BZL and ZX were involved in patient management. All authors read and approved the final manuscript.
